# Evidence of *Zip1* Promoting Sister Kinetochore Mono-orientation During Meiosis in Budding Yeast 

**DOI:** 10.1534/g3.118.200469

**Published:** 2018-09-25

**Authors:** Hemant Kumar Prajapati, Meenakshi Agarwal, Priyanka Mittal, Santanu K. Ghosh

**Affiliations:** * Department of Biosciences and Bioengineering, Indian Institute of Technology, Bombay, Powai, Mumbai-400076, India; † Department of Biological Science, Florida State University, Tallahassee, FL

**Keywords:** Synaptonemal complex, *Zip1*, mono-orientation, meiosis, budding yeast

## Abstract

Halving of the genome during meiosis I is achieved as the homologous chromosomes move to the opposite spindle poles whereas the sister chromatids stay together and move to the same pole. This requires that the sister kinetochores should take a side-by-side orientation in order to connect to the microtubules emanating from the same pole. Factors that constrain sister kinetochores to adopt such orientation are therefore crucial to achieve reductional chromosome segregation in meiosis I. In budding yeast, a protein complex, known as monopolin, is involved in conjoining of the sister kinetochores and thus facilitates their binding to the microtubules from the same pole. In this study, we report *Zip1*, a synaptonemal complex component, as another factor that might help the sister kinetochores to take the side-by-side orientation and promote their mono-orientation on the meiosis I spindle. From our results, we propose that the localization of *Zip1* at the centromere may provide an additional constraining factor that promotes monopolin to cross-link the sister kinetochores enabling them to mono-orient.

Conservation of ploidy during sexual reproduction depends on the successful generation of a gamete with genome content precisely half of its mother progenitor cell. The process through which this happens is meiosis which consists of one round of DNA replication followed by two rounds of chromosome segregations. During the first division of meiosis (meiosis I) the homologous chromosomes separate from each other and move to the opposite spindle poles. At the same time, the two sister chromatids that form each homologous chromosome, remain glued together by cohesin and move to the same pole. Meiosis I ends up with two nuclei each having ploidy half of the mother cell. The success of this reductional segregation of the chromosomes depends on three key meiosis I specific events. One, the pairing of the homologous chromosomes that culminates into recombination mediated physical linkage between the homologs. Second, the sister kinetochores attach to the microtubules emanating from the same spindle pole (mono-orientation) resulting in the sister chromatids to co-segregate and third, the cohesins at the centromere and pericentromere are protected from degradation.

 Although the overall process of meiotic chromosome segregation is conserved from yeast to mammals, there are variations in the mechanism of how the sister kinetochores are mono-oriented on meiosis I spindle. For instance, Rec8-cohesin is the key factor for such mono-orientation in *S. pombe* and higher eukaryotes (Chelysheva *et al.* 2005; Yokobayashi and Watanabe 2005), whereas the same is not true in *S. cerevisiae* (Tóth *et al.* 2000). In this organism a four protein complex termed monopolin is responsible for holding the two sister kinetochores together enabling them to connect to a single microtubule and thus to become mono-oriented with respect to the spindle pole (Marston and Amon 2004; Monje-Casas *et al.* 2007). Mam1 is the first protein of this complex that was discovered and is expressed only during meiosis I (Tóth *et al.* 2000). The other two proteins, Csm1 and Lrs4, are nucleolar proteins and become targeted to the centromere at the beginning of meiosis I (Rabitsch *et al.* 2003). The lastly discovered component is Hrr25, a casein kinase whose kinase activity is required for mono-orientation (Petronczki *et al.* 2006).

It is believed that monopolin acts as a molecular clamp to keep the sister kinetochores together (Corbett *et al.* 2010; Corbett and Harrison 2012; Sarangapani *et al.* 2014). Therefore, in principle, any condition that would favor less rotational freedom for the sister kinetochores may facilitate a side-by-side geometry of the sister kinetochores which then, in turn, would promote localization of monopolin at the centromere to do the final task of clamping. In fact, the role of condensin on mono-orientation of the sister kinetochores in different organisms may follow this notion (Brito *et al.* 2010; Tada *et al.* 2011; Burrack *et al.* 2013).

In this study, we wished to address if there are other factors that might constrain the chromosomes to adopt a side-by-side geometry of the sister kinetochores and we assume such a factor should act upstream of the kinetochore-microtubule attachment. The pairing of the homologs at early prophase is such an upstream event. The assembly of the synaptonemal complex (SC) along the length of the homologous chromosomes reinforces the pairing. The SC is a tripartite structure with two lateral and one central elements, and it holds the two homologs in near vicinity that favors homolog pairing. In *S. cerevisiae*, the assembly of SC initiates through programmed double-strand break made by Spo11 endonuclease (Henderson and Keeney 2004). The major component of SC, *Zip1* is necessary to tightly juxtapose the homologous chromosomes (Sym *et al.* 1993; Sym and Roeder 1995; Dong and Roeder 2000). Upon disassembly of SC toward the end of pachytene, the homologs remain joined to each other by reciprocal crossovers called chiasmata. *Zip1* appears at the centromere before SC assembly starts and then spread along the chromosomes as SC forms. This early localization of *Zip1* at the centromere causes a phenomenon called ‘centromere coupling’ where *Zip1* promotes pairing of the non-homologous chromosomes in a homology-independent manner (Tsubouchi and Roeder 2005; Falk *et al.* 2010). Interestingly, *Zip1* persists specifically at the centromere even after disassembly of SC until metaphase I, (Gladstone *et al.* 2009; Newnham *et al.* 2010), a time window that overlaps with the time when the sister kinetochores become attached unidirectionally to the microtubule (Miller *et al.* 2012). This extended localization of *Zip1* at the centromere is believed to facilitate bi-orientation of the homologs on the meiosis I spindle (Gladstone *et al.* 2009; Newnham *et al.* 2010). It is proposed that apart from the physical linkage between the homologs as chiasmata, localization of *Zip1* at the centromere constrains the homologous centromere pairs to take a back-to-back geometry so that each pair can attach to the microtubules from the opposite poles and become bi-oriented. 

Given the facts that *Zip1* is capable of bridging proteinaceous structure as it joins the lateral elements during SC formation and it can constrain the rotational freedom of the kinetochores by localizing specifically at the centromere, we wished to test if this protein has any similar role in cross-bridging sister kinetochores by constraining them to take a side-by-side geometry required for mono-orientation. To examine this, we analyzed the *zip1* deletion mutant for its role in sister kinetochore mono-orientation. From the results presented here, we propose that retention of *Zip1* at the centromere beyond SC disassembly and at the time of kinetochore-microtubule attachment, facilitates sister kinetochores staying together that in turn aids in Mam1 stability at the kinetochore and hence sister mono-orientation.

## Materials and Methods

### Yeast strains

All the yeast strains used in this study were of SK1 background. Detailed genotype for all the strains is mentioned in Table S1 of supplementary information. Deletion, tagging, and promoter shuffling of the genes were performed using PCR cassettes amplified from respective plasmids obtained from Euroscarf (Longtine *et al.* 1998; Janke *et al.* 2004). 

### Chromosome segregation assay

In order to fluorescently mark Chromosome V, a plasmid containing TetO repeats (224 copies) was integrated at 1.4 kb away from *CENV*, and GFP-TetR was expressed ectopically as described earlier (Michaelis *et al.* 1997). This marking could be either heterozygous (both the sisters of one homolog are marked) or homozygous (both the homologs are marked). Fluorescence microscope Zeiss Axio Observer.Z1 was used for live cell imaging as described earlier (Mehta *et al.* 2014; Prajapati *et al.* 2017). In all the cell biology experiments, cell counting was performed at least for two times, and the error bars represent the standard deviation from the mean.

### Meiotic progression

Meiotic synchronization and progression of the cell cycle were performed as described earlier in details (Mehta *et al.* 2014). A single colony of respective yeast strain was inoculated in 5 ml of YPD (yeast extract 1%, peptone 2%, and dextrose 2%) broth and was grown for overnight. Cells were diluted to O.D._(600)_=0.2 in 25 ml of YPA (1% yeast extract, 2% peptone, 1% potassium acetate) and were grown for 12 to 16 h. Further, cells of O.D._(600)_=1.6-1.8, were transferred to 25 ml of sporulation media, SPM (potassium acetate 0.3%, raffinose 0.02%, supplemented with a 1/4^th^ concentration of the auxotrophic amino acids). Cells were harvested at different time points for immunofluorescence and live cell imaging.

### Sporulation efficiency and spore viability

As described above, cells were progressed into meiosis in the SPM media until sporulation (24 h). Samples were observed under a microscope for finding a total number of sporulated cells (having either 1,2,3 or 4 spores) which was divided with a total number of examined cells to calculate the sporulation efficiency as explained previously (Mehta *et al.* 2014). Spores were planted on YPD plate using Zeiss Scope.A1 microscope and calculation of germinated spores out of the total number of spores planted was denoted as percentage spore viability. Spore viability was calculated for two times with 30 tetrads dissected each time.

### Indirect immunofluorescence and chromatin spread

 Indirect immunofluorescence and Chromatin spread were performed as described previously in details (Mehta *et al.* 2014; Agarwal *et al.* 2015). Typically, the spheroplasted cells were incubated with blocking solution (5% skim milk prepared in 10 mg/ml BSA with Phosphate Buffered Saline) for 15 min, and after washing of the cells, primary antibodies (rat anti tubulin, YOL1/34, Serotech, UK, 1: 5000; rat anti HA 3F10, Roche, Germany, 1:200; mouse anti myc 9E10, Roche, Germany, 1:200; mouse anti GFP, Roche, Germany, 1:200) were added and kept for 1 h. After 3-4 washes with PBS, cells were incubated with appropriate secondary antibodies (TRITC-conjugated goat anti-rat, 112-025-167, Jackson ImmunoResearch Laboratories, USA, 1:200; Alexa flour 488-conjugated goat anti-mouse, 115-545-166, Jackson ImmunoResearch Laboratories, USA, 1:200). After 3-4 washes DAPI (1µg/ml) was added followed by mounting of the slides. 

### ChIP assay

Chromatin immunoprecipitation assay was performed as discussed earlier in details (Prajapati *et al.* 2017). Typically, the wild-type and *zip1*Δ strains were grown in YPA for around 14 to 18 h and were transferred to SPM for a synchronized meiotic progression. The samples were collected from 5 to 8 h in every one and a half hour interval for chromatin spread and Mam1-9Myc localization was examined. The Mam1 signal was clearly observed at 5 and 6.5 h in the wild-type and in the *zip1*Δ cells, respectively (Figure S4). Therefore, the cells were harvested at these respective time points to perform either ChIP assay (for the Mam1-9Myc association to the centromere) or co-localization study (for Mam1-9Myc and Ndc10-6HA) in the wild-type and *zip1*Δ strains. For ChIP assays, around 5x10^8^ cells were fixed using 1% formaldehyde for 1 h. After lysis of the cells by glass beads, chromatin was sheared to around 200-500 bp using probe sonicator (21 s on, 1 min on ice, 12 cycles) in total 400 µl volume of lysis buffer (50 mM HEPES-KOH, pH 7.5, 140 mM KCl, 1 mM EDTA, 1% Triton X-100, 0.1% sodium deoxycholate, freshly added protease inhibitor cocktail from Roche, Germany). The lysate was cleared at 20000 g for 15-20 min; the supernatant was divided into three tubes as follows- 50 µl for Input/WCE (Whole Cell Extract), 150 µl each for +Ab and –Ab samples (250 µl lysis buffer was added to make up the volume till 400 µl). The anti myc (for Mam1-9Myc) antibodies (rabbit polyclonal, ab9106, 2 µgm/sample, Abcam) were added in +Ab samples. After 4 h incubation at 4° in rotating condition, protein A sepharose beads were added and kept in rotation for 1 h. Beads were washed and DNA was purified from all the samples after decrosslinking as previously described (Prajapati *et al.* 2017). The qPCR reaction was performed using Agilent Technologies MX3000P real-time PCR machine in SYBR green reaction mixture and the Ct values were imported from MxPro v4 10d Software (Agilent). To find out enrichment/input the ΔCT was calculated as mentioned earlier (Mehta *et al.* 2014; Verzijlbergen *et al.* 2014), ΔCT= Ct (ChIP) – [Ct (Input) – logE (Input dilution factor)], here E represents specific primer efficiency. The final enrichment/input value was obtained by E^^- ΔCT^. The ChIP experiment was performed for at least three times and the error bars represent the standard deviation from the mean. Primer sequences used for qPCR are mentioned in Table S2.

### Other methods

For removal of the microtubules during the meiotic progression of *spo11*Δ, *zip1*Δ *spo11*Δ and *mam1*Δ *spo11*Δ strains, 100 µg/ml benomyl was added following 6 h of their release into SPM medium and the cultures were incubated for further 2 h before harvesting. Before the addition of benomyl, the cells were analyzed for the spindle length by immunofluorescence using anti tubulin antibodies to verify the metaphase I stage. The microtubules were depolymerized subsequently by addition of benomyl (Figure S3, Hochwagen *et al.* 2005). 

To analyze the co-localization between Mam1 and Ndc10, the ‘Imaris coloc tool’ was utilized to calculate the Pearson’s correlation coefficient (Adler and Parmryd 2010). The detailed description of the method has been described elsewhere (Figure S9 of Prajapati *et al.* 2017).

In order to analyze the DAPI staining (Figure 2A) distributed in 3D space, the cell images were captured at multiple planes using ‘z-stack’ tool of Zeiss Axio-vision software using motorized Axio Observer.Z1 microscope from Zeiss. For each image, the appropriate planes (where the fluorescence is bright and focused) were selected and merged to get an image in 2D and that was used for the qualitative measurement of DAPI stain.

### Data availability

Yeast strains are available upon request. The supplementary information file is available at FigShare. This file includes Tables S1, S2 and figures from S1 to S5. Supplemental material available at Figshare: https://doi.org/10.25387/g3.7105220.

## Results

### zip1Δ mutant shows defect in meiosis with an increased percentage of dyads

**Figure 1:**
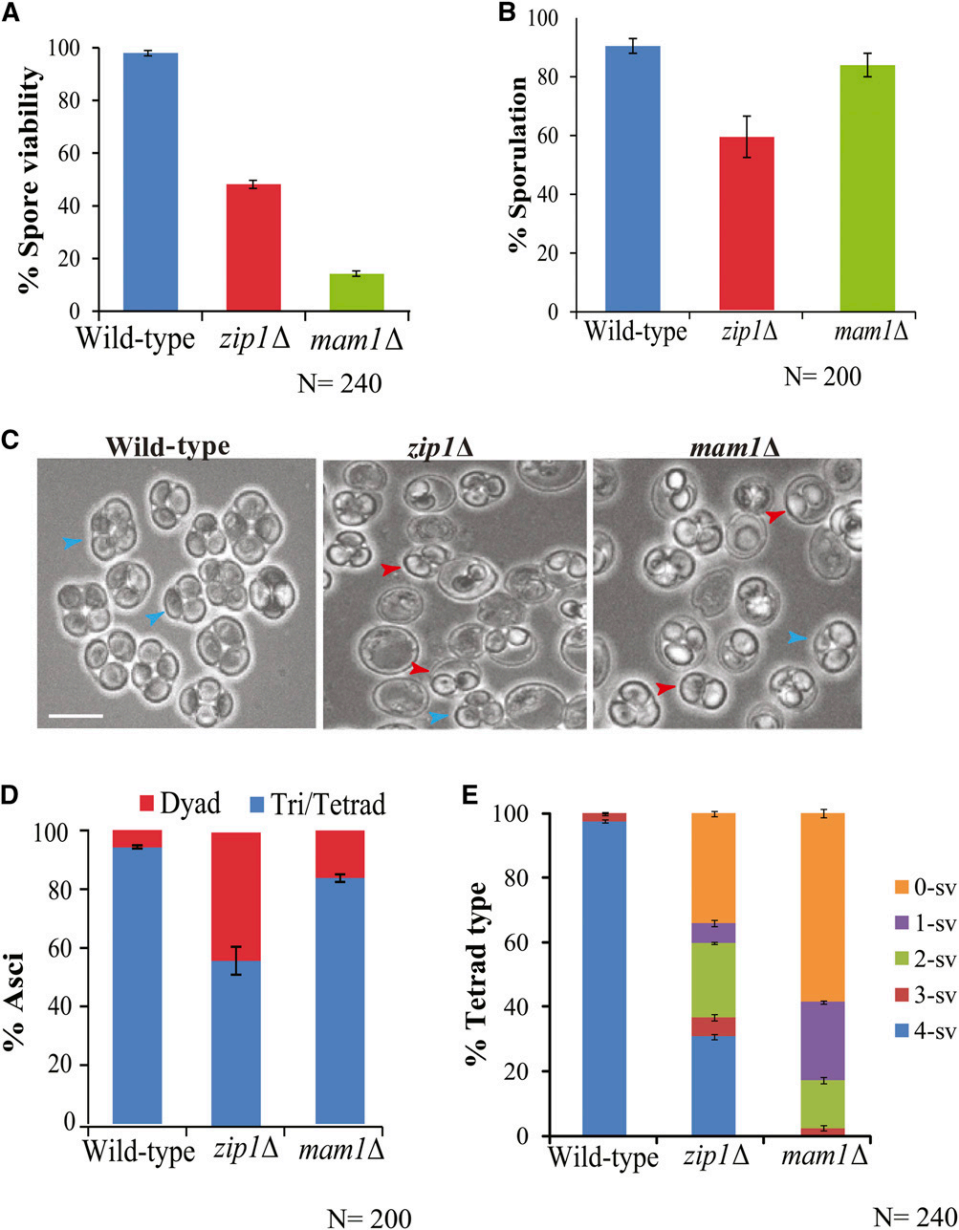
Deletion of *Zip1* causes reduced spore viability and increased dyads. (A) Spore viability assay in wild-type (SGY40), *zip1*Δ (SGY1066), and *mam1*Δ (SGY223). (B) Sporulation efficiency was determined by counting 200 cells for each strain in two independent experiments. (C) Field view of the sporulation cultures of the above strains after 24 h in SPM medium. Blue, and red arrowheads show tetrad/triad and dyads, respectively. Bar, ∼5 µm. (D) Distribution of types of asci in the above strains. Sporulation efficiency and type of asci were calculated after repetition of the experiments two times. (E) The percentage of tetrad type represents tetrads with 4, 3, 2, 1 and 0 spores viable (4-sv, 3-sv, 2-sv, 1-sv and 0-sv). % tetrad type was calculated from total 60 tetrads dissected for each strain. The spore viability and the percentage of tetrad type were calculated after tetrad dissection for two times (30 dissected in each time). Error bars represent the standard deviation from the mean.

Earlier studies have demonstrated the roles of *Zip1* in centromere coupling and the formation of SC (Sym *et al.* 1993; Dong and Roeder 2000; Tsubouchi and Roeder 2005; Falk *et al.* 2010). In this study, we wish to test its function in mono-orientation of sister chromatids during meiosis I. Therefore, as the first set of experiments we determined the spore viability of *zip1*Δ mutant and compared that with the wild-type and *mam1*Δ strains. While the wild-type strain showed 98%, *zip1*Δ and *mam1*Δ mutants showed ∼48% and ∼14% spore viability, respectively (Figure 1A) as reported earlier (Sym and Roeder 1994; Tóth *et al.* 2000; Bhuiyan and Schmekel 2004). Further, *zip1*Δ strain showed a reduced sporulation efficiency (59%) as reported earlier (Chen *et al.* 2015) as compared to both *mam1*Δ (84%) and wild-type (91%) strains (Figure 1B). Importantly, analysis of types of sporulation showed that *zip1*Δ gave more dyads asci (42%) than *mam1*Δ (15%) and wild-type strains (5%) (Figure 1C and D). Further, among the tetrads, we calculated the percentage of tetrads types on the basis of a number of viable spores from each tetrad (Figure 1E) and found 0- or 1-spore viable tetrads were more in the *mam1*Δ mutant. This result showed that generation of aneuploid spores was more in case of *mam1*Δ than *zip1*Δ mutant accounting for low spore viability for the former.

Generation of dyads is a hallmark feature of a cell compromised in sister chromatid mono-orientation with intact cohesin where meiosis I division is abrogated, and the cell enters into meiosis II. The spores obtained from the dyads are likely to be diploids (non-maters), and they will be mostly inviable due to aneuploidy as all the sisters will not be bi-oriented. A similar occurrence of dyads was observed in *spo13*Δ, or in *mam1*Δ mutants harboring sister mono-orientation defect and where meiosis I division is bypassed (Rutkowski and Esposito 2000; Tóth *et al.* 2000). However, although we observed an increased occurrence of dyads in *zip1*∆, the spores of those dyads were found to be haploids as judged by the zygote formation after mating with the tester strains (the known *MAT* a and *MAT* α strains) and these were less viable than the spores obtained from *mam1*Δ (Figure S1A). In *mam1*Δ cells, in the absence of mono-orientation, bi-orientation of the sisters leads to abrogation of meiosis I. Whereas in *zip1*∆ cells, the defects in SC and cross-over formation (Sym *et al.* 1993) and in homolog bi-orientation (Gladstone *et al.* 2009; Newnham *et al.* 2010) may result in either meiosis I abrogation or meiosis I homolog non-disjunction. This following normal meiosis II can cause the genome to be packaged into two spores with high inviability of the spores. However, the possibility for an additional defect in mono-orientation of the sister chromatids in *zip1*∆ cells cannot be ruled out with these experiments.

In order to examine if the dyads formation in *zip1*Δ strain, is either due to the starvation condition and delayed prophase as described previously (Neiman 2005) or linked to kinetochore orientation defect, we used the *zip1*Δ *spo11*Δ double mutant which does not show arrest at prophase (Obeso and Dawson 2010; Thacker *et al.* 2014). After sporulation, we observed around 20% dyads (Figure S1B), which is approximately half of the dyad population observed in *zip1*Δ alone which indicates a possible role of *Zip1* in kinetochore orientation. 

The previous study has shown that the cells without Mam1 take a longer time in meiosis I due to sister chromatid orientation defect and the cells arrest transiently at metaphase I (Tóth *et al.* 2000). If the *zip1*∆ cells, like *mam1*∆, show an orientation defect, it is expected that the cells will spend more time in metaphase I. However, *zip1*∆ cells arrest at meiotic prophase before a kinetochore-microtubule attachment takes place and this arrest can be abrogated by removing chiasmata (Sym *et al.* 1993; Obeso and Dawson 2010; Thacker *et al.* 2014). Therefore, to investigate if the *zip1*∆ cells, like *mam1*∆, can halt transiently in metaphase I, we followed the kinetics of meiotic progression of the *zip1*∆ cells in presence or absence of Spo11 which is required to generate DSB, a prerequisite for chiasmata formation (Figure S1C). Both *mam1*Δ and *zip1*Δ mutants were found to progress more slowly through meiosis I than the wild-type. As expected, *mam1*Δ cells showed a transient arrest at metaphase I (Figure S1C, the arrow on the blue line) and a significant population of *zip1*Δ cells showed prolonged arrest at prophase. Notably, around 14% cells of *zip1*Δ alone showed arrest at metaphase I (Figure S1C, the arrow on the red line) while rest remained arrested at prophase. However, when we followed the progression of *zip1*Δ *spo11*Δ double mutant, we failed to observe any transient arrest at metaphase I. We speculate that even if the sister kinetochores may bi-orient in the absence of *Zip1*, the kinetochore-microtubule amphitelic connection may not be robust enough to elicit a transient metaphase I arrest.

### zip1Δ cells show missegregation of the chromosomes similar to mam1Δ cells

**Figure 2:**
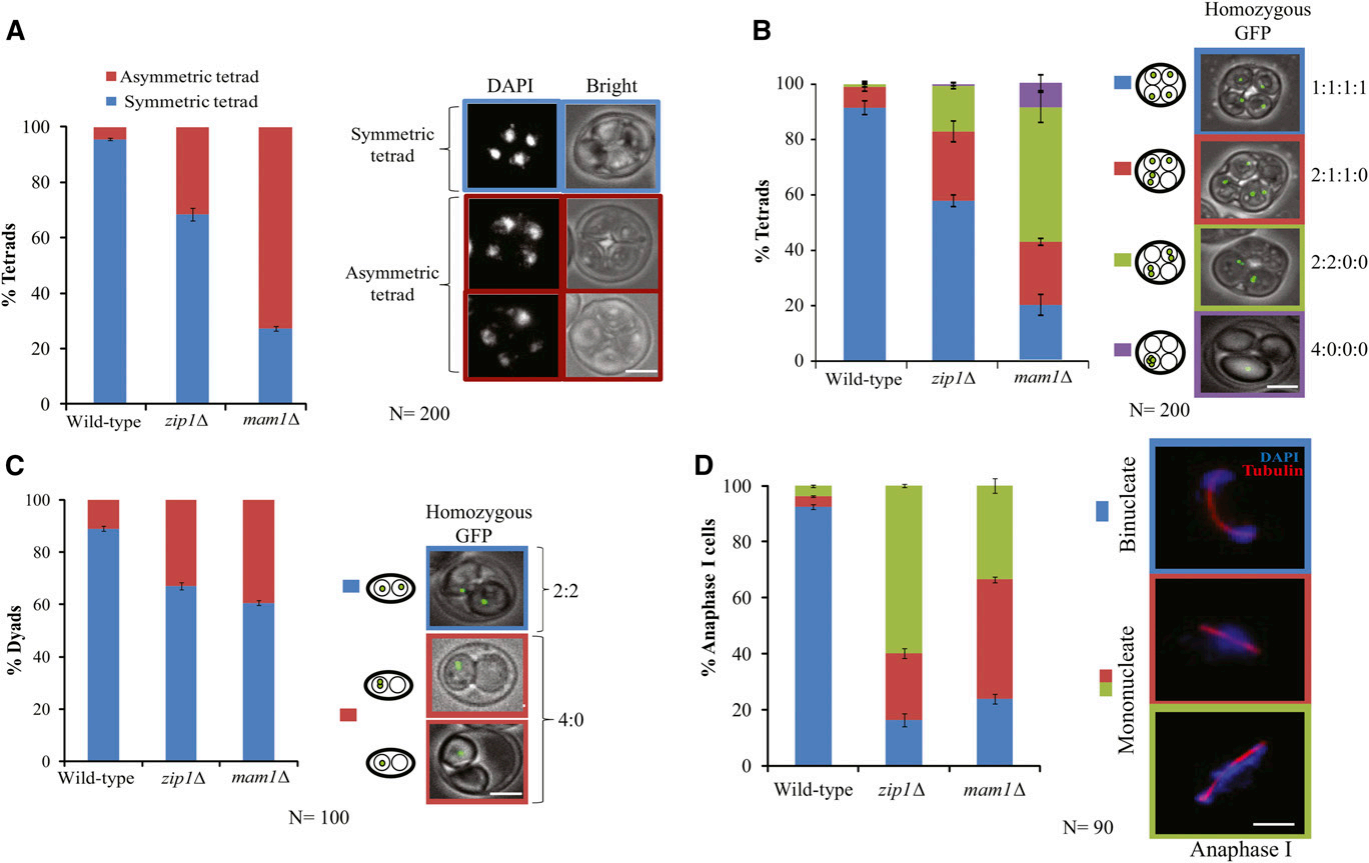
*zip1*Δ mutant shows chromosome segregation defects similar to *mam1*Δ. (A) The histograms show the percentage of asymmetric and symmetric tetrads in wild-type (SGY116), *mam1*Δ (SGY223) and *zip1*Δ (SGY1127) strains. (B) Segregation pattern of homozygous GFP dots in the above strains. (C) Segregation pattern of homozygous GFP dots in the dyads of *mam1*Δ and *zip1*Δ strains. (D) Immunofluorescence of the above strains showing the percentage of anaphase I cells harboring mono- or bi-nucleates. DNA and spindle were visualized using DAPI and anti-tubulin antibodies, respectively. The absence of *Zip1* gives an equal number of mono-nucleates like *mam*1Δ at the end of meiosis I. Bar, ∼2 µm. Two independent experiments were performed for each strain, and the average values are shown with a total number of cells represented by N. Error bars represent the standard deviation from the mean.

Reduced spore viability in *zip1*Δ suggests that many of the generated spores obtaining from the tetrads population are aneuploids (Figure 1A and E). DAPI staining of the tetrads showed that this is true where around 31% of the tetrads were asymmetric in *zip1*Δ strain compared to only 5% in the wild-type (Figure 2A), whereas in *mam1*∆ around 72% cells showed asymmetric tetrads. In such tetrads, two nuclei had more DNA than the other two that might have caused due to a partial abrogation of meiosis I segregation (not for all chromosomes) due to sister chromatids bi-orientation and their non-disjunction due to retention of cohesion. The presence of less percentage of asymmetric tetrads in *zip1*∆ than in *mam1*∆, suggests that lack of *Zip1* might cause fewer pairs of sister chromatids to bi-orient than in the cells lacking Mam1. However, the generation of asymmetric tetrads in *zip1*∆ cells may additionally occur due to perturbation in SC or crossover formation causing meiosis I non-disjunction. Nevertheless, these tetrads contain aneuploid spores resulting in less than 50% spore viability in *zip1*Δ cells (Figure 1A and E).

To further ascertain the fact that the generation of asymmetric tetrads is a consequence of the completion of meiosis II without any meiosis I, we followed the segregation of the homologs by labeling them with fluorescence. For this, we marked chromosome V with GFP-TetR/TetO system (Michaelis *et al.* 1997) in the homozygous condition in the wild-type, *zip1*Δ, and *mam1*Δ cells and analyzed the segregation of the four GFP dots each depicting one copy of chromosome V (Figure 2B). Faithful chromosome segregation during meiosis results in four nuclei each having one of the four marked sister chromatids (one GFP dot) and termed as 1:1:1:1 pattern of chromosome segregation (Figure S2A). Whereas, any defect due to lack of homolog pairing, homolog bi-orientation and/or mono-orientation of sister chromatids will generate tetrads with 2:1:1:0, 2:2:0:0 and 4:0:0:0 patterns (Figure S2B, C and D). Lack of homolog pairing will lead the homologs to segregate randomly in meiosis I that may result in 2:2 or 4:0 pattern in the bi-nucleates at the end of meiosis I which will turn into 1:1:1:1 or 2:2:0:0 pattern, respectively at the end of meiosis II. However, additional meiosis II non-disjunction will lead to 2:1:1:0 or 4:0:0:0 patterns, respectively. On the other hand, lack of mono-orientation in all the sister pairs will lead to no segregation of chromosomes in meiosis I followed by disjunction or non-disjunction in meiosis II to produce binucleates with 2:2 or 4:0 patterns, respectively. However, if only a subset of the sister pairs bi-orient, that will lead to an overall segregation of the chromosomes in meiosis I keeping the bi-oriented pairs at the middle and such cells upon entering into meiosis II will give 2:2:0:0 or 2:1:1:0 (if disjoined) and 4:0:0:0 (if non-disjoined) patterns (Figure S2C and D). Therefore, to judge the type of defect that *zip1*∆ strain might harbor in addition to known homolog pairing and bi-orientation defects, we compared the GFP dots distribution between *mam1*Δ and *zip1*Δ strains. Similar frequencies of 2:1:1:0 (∼25%) pattern was observed both in *mam1*Δ and *zip1*∆ mutants (Figure 2B) suggesting meiosis II non-disjunction occurs in both these strains. As expected *mam1*∆ showed a high percentage (48%) of 2:2:0:0 segregation accounting for lack of sister mono-orientation and abrogation of meiosis I. Interestingly, *zip1*Δ mutant also showed a moderate percentage (16%) of 2:2:0:0 segregation which may be due to both random segregation of the homologs in meiosis I due to failure in homolog pairing and bi-orientation as well as due to the abrogation of meiosis I upon bi-orientation of sisters on the meiosis I spindle. Wild-type cells, as expected, mostly showed 1:1:1:1 pattern of segregation.

If the observed percentage of dyads (Figure 1D) in *zip1*∆ and *mam1*∆ cells is due to complete abrogation of meiosis I, then normal meiosis II segregation will result in 2:2 segregation of homozygous GFP dots and any non-disjunction during the process will lead to 4:0 segregation. Remarkably, both *mam1*Δ and *zip1*Δ gave almost similar 2:2 and 4:0 patterns of GFP dot distribution among the dyads (Figure 2C) suggesting similar types of defects, presumably sister bi-orientation during meiosis I, occurring in these mutants.

### zip1Δ mutant produces mono-nucleated cells similar to mam1Δ cells

From the appearance of dyads in the *zip1*Δ cells (Figure 1D), and the patterns of DAPI and GFP segregations in the dyads and the tetrads (Figure 2A-C), much like that of *mam1*Δ cells, it can be tempting to presume that *Zip1* might have a role in mono-orienting sister chromatids. To test this further, we hypothesized that if mono-orientation is suppressed in the absence of *Zip1*, each pair of the sister chromatids will be pulled from opposite spindle poles. However, the cohesin present in between the sisters will restrict the separation resulting blockage of meiosis I chromosome segregation. Under this condition, the cells will remain as mono-nucleated but will proceed biochemically to elongate the spindle giving anaphase I spindle. This will subsequently proceed to complete meiosis II with the single nuclear division. This phenotype was previously reported in *mam1*∆ cells (Tóth *et al.* 2000).

To assess whether *zip1*∆ mutant can suppress mono-orientation and produce mono-nucleates, we analyzed the cells toward the end of meiosis I with anaphase I spindle. This excludes the *zip1*∆ cells that become arrested just after pachytene (Sym *et al.* 1993). Both *zip1*Δ and *mam1*Δ mutants showed ∼75% of mono-nucleated cells as compared to only 5% in the wild-type strain (Figure 2D). A high percentage of mono-nucleated cells in *mam1*∆ cells at the end of meiosis I, consistent with the earlier report (Tóth *et al.* 2000), occurs due to higher suppression of mono-orientation of the sister kinetochores. On the other hand, the similar high percentage of mono-nucleated cells in *zip1*∆ cells may result from lack of homolog bi-orientation (Gladstone *et al.* 2009) and/or suppression of mono-orientation of the sister kinetochores related to the function of this protein at the centromere. Lack of bi-orientation with poor SC formation can disperse the homologs at different positions between the two SPBs in metaphase I and upon anaphase I onset the homologs, and hence the DAPI can be found ‘distributed’ along the anaphase I spindle (Figure 2D, green shade). Whereas if the sisters are bi-oriented in metaphase I, they will remain at the middle of the two SPBs and can produce a ‘roundish’ DAPI mass on anaphase I spindle (Figure 2D, red shade). To distinguish these two categories, we observed the mono-nucleated cells arising in *mam1*∆ or *zip1*∆ mutants carefully. Out of the mono-nucleated cells in *mam1*∆, around 42% showed ‘roundish’ nuclei accounting for predominant suppression of mono-orientation. Whereas in *zip1*∆, the majority of the mono-nucleated cells (60%) showed ‘distributed’ DAPI (Figure 2D, green shade) indicating a defect in homolog bi-orientation and imperfect SC formation. Importantly, a sizable fraction of mono-nucleated cells (23%) also showed ‘roundish’ nuclei (Figure 2D, *zip1*∆ category, red shade) suggesting the occurrence of suppression of mono-orientation.

### *Zip1* facilitates mono-orientation of the sister kinetochores

**Figure 3:**
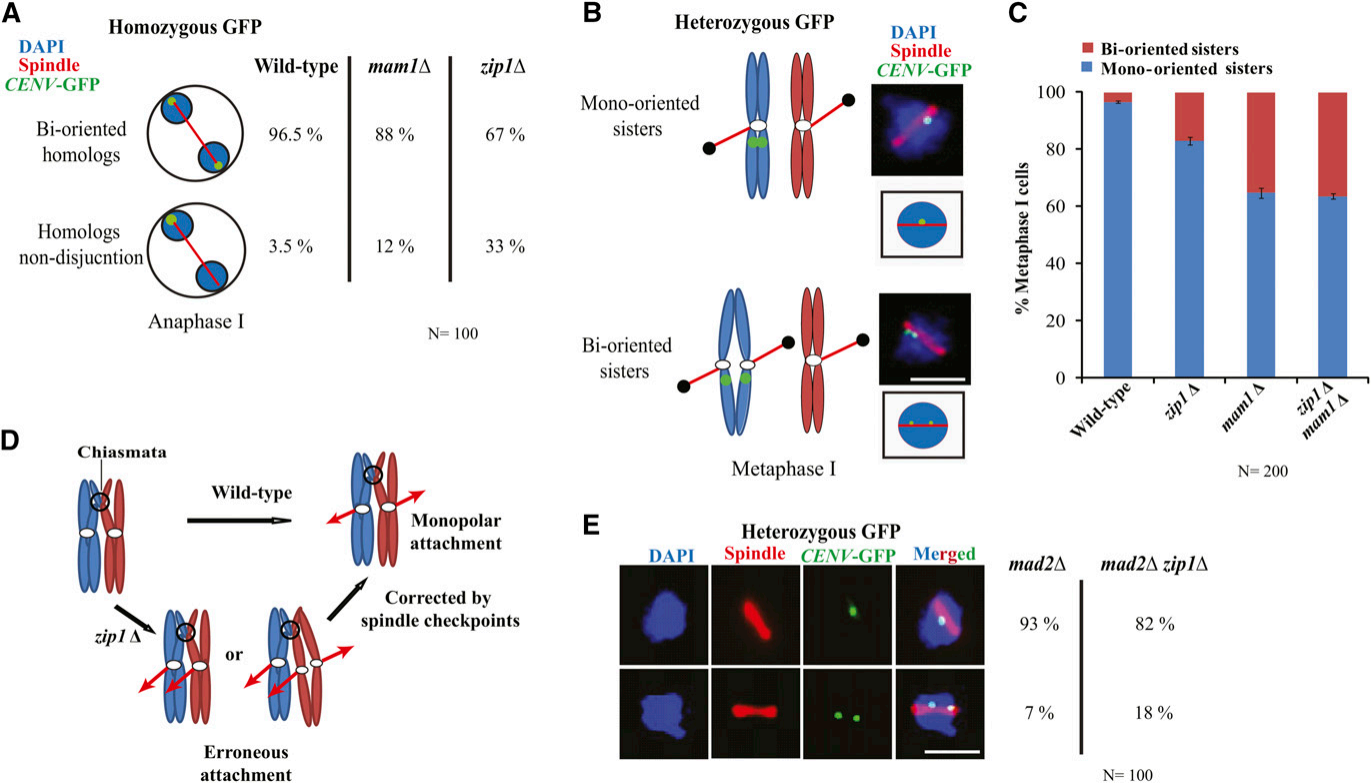
The absence of *Zip1* causes homologs non-disjunction and sister chromatid bi-orientation during meiosis I. (A) Immunofluorescence assay is showing the percentage of anaphase I cells of wild-type (SGY116), *mam1*Δ (SGY223) and *zip1*Δ (SGY1127) with homologs disjunction or non-disjunction. Around 100 cells were analyzed for each strain. (B) Schematic of mono or bi-orientation of sister chromatids along with the representative images during metaphase I (C) Immunofluorescence analysis showing the percentage of wild-type (SGY115), *mam1*Δ (SGY220), *zip1*Δ (SGY1427) and *mam1*Δ *zip1*Δ (SGY3190) cells at metaphase I with mono- or bi-oriented sister chromatids. The cells were counted from two independent experiments for each strain and the total number of cells are represented as N. (D) Schematic showing monopolar attachment of the sister chromatids during metaphase I and possible correction of erroneous attachment by spindle checkpoint proteins. (E) The mono-oriented and bi-oriented sisters were counted similarly as described in ‘B’ using *mad2*Δ (SGY1265) and *zip1*Δ *mad2*Δ (SGY1267) cells. Around 100 cells were observed for each strain. Bar, ∼5 µm. Error bars represent the standard deviation from the mean.

Previous studies have demonstrated that an early localization of *Zip1* at the centromere is required for ‘centromere coupling’ (Tsubouchi and Roeder 2005; Falk *et al.* 2010). This is followed by its role in homolog pairing and SC formation. Interestingly, *Zip1* remains at the centromere even after SC disassembly and this extended localization of *Zip1* at the centromere promotes bipolar attachment of the homologous chromosomes (Gladstone *et al.* 2009) and the non-exchange chromosomes to the spindle (Newnham *et al.* 2010). To confirm this, we marked both the homologs of chromosome V with GFP-TetR/TetO (Michaelis *et al.* 1997) and observed the anaphase I cells of the wild-type, *mam1*Δ, and *zip1*Δ. As expected *zip*1Δ mutant showed an increased percentage of homolog non-disjunction (∼33%) as compared to the wild-type (3.5%) and *mam1*Δ (∼12%) strains (Figure 3A) suggesting, defects in bipolar attachment of the homologs, and our *zip1*Δ mutant conforms to the earlier report (Gladstone *et al.* 2009).

The role of *Zip1* in bi-orientation of the homologs, perhaps by constraining the homologs (Gladstone *et al.* 2009; Newnham *et al.* 2010), may also principally facilitate the sister chromatids to mono-orient. Increased frequency of occurrence of dyads (Figure 1D), 2:2:0:0 segregation of the homozygous GFP dots (Figure 2B) and mononucleated cells with anaphase I spindle (Figure 2D) in the *zip1*Δ cells also suggest that *Zip1* might possess a moderate role in mono-orientation of the sister kinetochores on metaphase I spindle. Therefore, to directly visualize the sister kinetochore orientation on metaphase I spindle, we marked only one copy of the homolog (only one pair of sister chromatids) with GFP-TetR/TetO at *CENV* in the wild type, *mam1*Δ, and *zip1*Δ cells. In the wild-type, sister chromatids remain cohesed and mono-oriented during metaphase I and appear as a single GFP dot on the spindle whereas, upon bi-orientation sister chromatids will be pulled from the opposite poles causing them to split and the sisters will appear as two split GFP dots on the spindle (Figure 3B). Therefore, to distinguish single *vs.* two split GFP dots, cells were released into synchronized meiosis. As discussed above, a majority of *zip1*Δ cells showed strong delay at pachytene/prophase (Figure S1C). However, a population of cells (14%) instead arrested at metaphase I. We counted the GFP dots in those metaphase I cells as judged by mono-nucleates with short spindles. A fraction of *zip1*Δ nuclei (17%) showed two split GFP dots which are more than thrice as compared to its isogenic wild-type (4%, Figure 3C). Whereas 35% of *mam1*Δ nuclei, used as a control, showed such splitting indicating bi-orientation of the sister chromatids on metaphase I spindle which is consistent with an earlier report (Kiburz *et al.* 2008, Figure 3C). Our observed frequency (17%) of two split GFP dots in *zip1*∆ mutant may be an underestimate. This is because earlier studies have shown that spindle checkpoint proteins can potentially correct erroneous attachment, if any, during metaphase and can lead the cell toward faithful chromosome segregation (Gillett *et al.* 2004). This correction becomes possible in a spindle checkpoint dependent way in the *zip1*Δ cells as they, besides prophase arrest, also arrest transiently at metaphase I when the kinetochore-microtubule attachment becomes established (Figure 3D, Sym *et al.* 1993; Bailis *et al.* 2000; Gladstone *et al.* 2009). Therefore, we thought that this correction could be the reason why the *zip1*Δ mutant did not show a higher frequency of sister bi-orientation. Hence, we wished to assess the mono-orientation defect in the *zip1*∆ cells devoid of any error correction mechanism. For this, we repeated the sister chromatid mono-orientation assay in *zip*1∆ cells deleted for spindle checkpoint gene *MAD2*. However, the *zip1*∆ *mad2*∆ double mutant showed a similar frequency of two split GFP dots (18%, Figure 3E) compared to *zip1*∆ (17%, Figure 3C) but more than *mad2*∆ alone (7%, Figure 3E).

**Figure 4:**
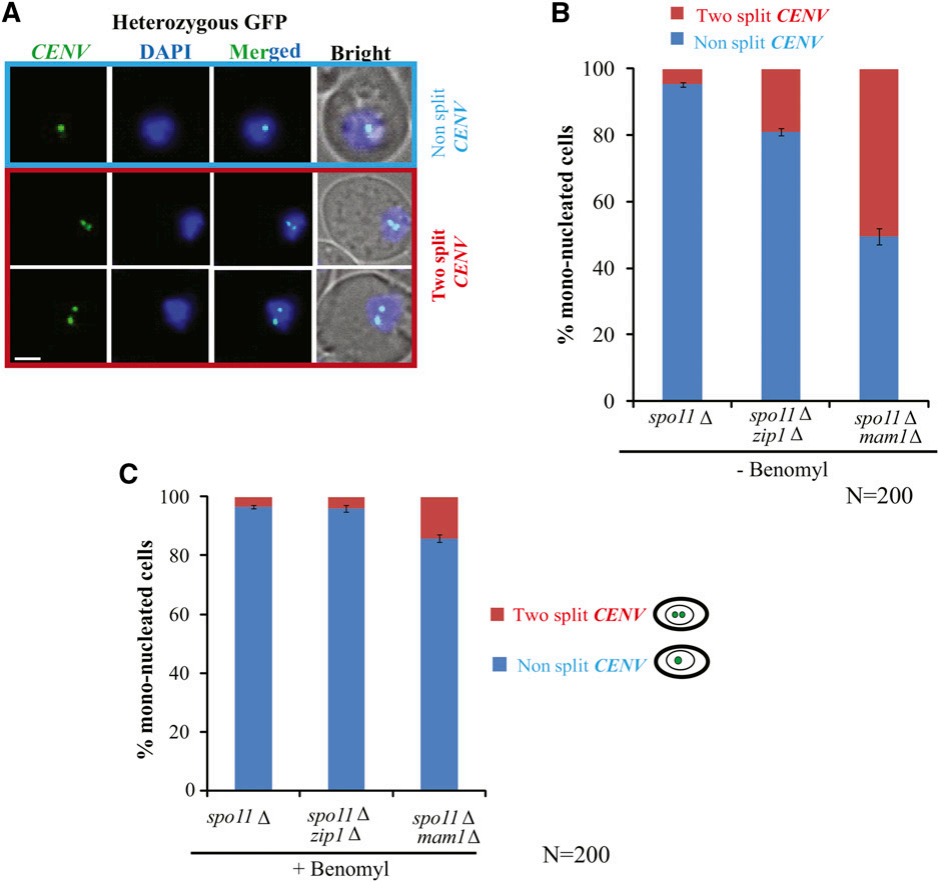
Sister chromatids bi-orientation in *zip1*Δ is independent of SC formation but requires microtubule mediated pulling. (A and B) Live cell imaging showing two split *vs.* non split *CENV*-GFP dots in *spo11*Δ (SGY3180), *zip1*Δ *spo11*Δ (SGY3179) and *mam1*Δ *spo11*Δ (SGY3178) cells. The mono-nucleated cells were scored for the GFP dots after transferring the cultures into SPM for 8 h. (C) The same experiment was performed where the cells were treated with 100 µgm/ml benomyl for 2 h after 6 h of culturing into SPM and many of the cells were in metaphase I before addition of the drug. The cells were counted from two independent experiments for each strain and the total numbers of cells are represented as N. Error bars represent the standard deviation from the mean. Bar, ∼2 µm.

To negate the possibility that the increased two split GFP dots in the *zip1*∆ cells over the wild-type is due to a role of *Zip1* in SC formation, we performed the above mono-orientation assay in the *zip1*∆ cells where *SPO11* was also deleted. The *spo11*Δ alone and the *mam1*Δ *spo11*Δ double mutants were used as controls. We have analyzed the mono-nucleated cells after 8 h in SPM and examined the GFP dot segregation among all the strains (Figure 4A). We found that ∼50% mono-nucleated cells showed two split GFP dots which reflects the bi-orientation of sister chromatids in *mam1*Δ *spo11*Δ strain. However, around ∼18% of cells showed this phenomenon in the *zip1*Δ *spo11*Δ strain in comparison to 4–5% of *spo11*Δ cells (Figure 4B). This result indicates that the observed defect in mono-orientation is not due to lack of SC formation in the *zip*1∆ cells.

The observed splitting of two GFP dots in the *zip1*∆ cells may occur due to a cohesion defect between the two sisters. To examine this, the pulling force from microtubules was removed by depolymerization of microtubules so that any sister separation will account only for the cohesion defect but not for any bi-orientation. Removal of microtubules caused a large reduction in the frequency of two split GFP dots in all the strains (Figure 4C) indicating that the splitting of the sister centromeres occurs predominantly due to bi-orientation of the sisters. This also suggests that the data presented in figure 2B (the 2:2:0:0 segregation pattern) may not be due to cohesion defect between the sister chromatids. Depolymerization of the microtubules was confirmed by immunofluorescence using anti-tubulin antibodies (Figure S3).

Overall, the above results indicate that the lack of *Zip1* causes bi-orientation of the sister chromatids, albeit at a moderate level but significantly at a frequency which is more than thrice to the wild-type. To address if the mono-orientation function of *Zip1* is independent of Mam1, we investigated the percentage of two split GFP dots in *zip1*∆ *mam1*Δ double mutant strain. However, we found no synthetic enhancement of the defect in *zip1*∆ *mam1*Δ as opposed to *mam1*Δ (Figure 3C) cells suggesting a common pathway for the functioning of *Zip1* and Mam1 in sister chromatid mono-orientation.

### The localization of Mam1 at the centromere is reduced in the absence of *Zip1*

**Figure 5:**
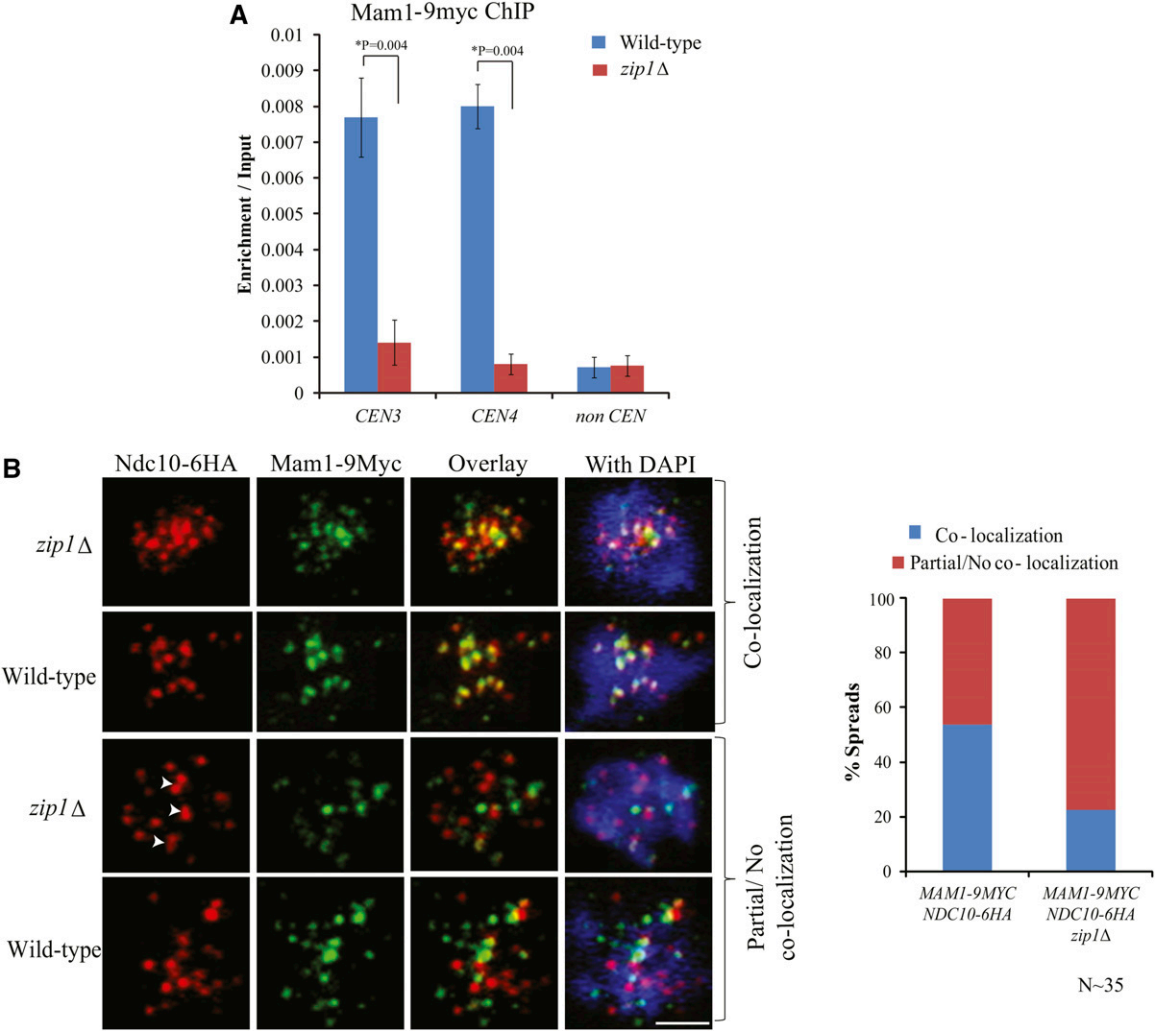
Mam1 is mislocalized in the absence of *Zip1*. (A) ChIP assay for quantifying the association of Mam1-9Myc at the centromeres in the presence or absence of *Zip1*. Anti myc antibodies were used to pull down Mam1-9Myc. The experiment was repeated for three times and error bars represent the standard deviation from the mean. *The P value was determined by using ‘two tailed *t*-test assuming unequal variances’ option from the data analysis tool of Microsoft Excel. (B) Chromatin spread showing localization of Mam1-9Myc and Ndc10-6HA in prophase/metaphase I cells of wild-type (SGY1444) and *zip1*Δ (SGY1410). Arrowhead shows doublet of Ndc10 foci that signifies kinetochores remained together by flanking crossover in the absence of *Zip1* (Gladstone *et al.* 2009). On the basis of the localization of Mam1-9Myc with respect to Ndc10-6HA, the cells were divided into two categories: co-localization, partial or no co-localization. Bar, ∼5 µm.

From the above results, it is evident that *Zip1* facilitates the mono-orientation of the sister chromatids through the Mam1 pathway. We speculate that *Zip1* does this by holding the sister kinetochores in the near vicinity which facilitates Mam1 to bridge the sisters by binding to the kinetochores through Dsn1 subunits (Sarkar *et al.* 2013). Therefore, we wished to test the localization of Mam1 at the centromere in the absence of *Zip1* in cells arrested at metaphase I. We found a high perturbation in Mam1 localization at the centromere in the absence of *Zip1* compared to the wild-type using ChIP assay (Figure 5A). Chromatin spreads were also performed from the same cells to investigate the localization of Mam1 at the centromeres that were marked by the inner kinetochore protein Ndc10 (Figure 5B and Figure S4). The Pearson’s correlation coefficient was measured using Imaris ‘coloc’ tool in order to find out the significant colocalization between Ndc10-6HA and Mam1-9Myc foci as previously described (Zinchuk and Zinchuk 2008; Adler and Parmryd 2010; Prajapati *et al.* 2017). We detected a significant mislocalization of Mam1 in the *zip1*∆ mutant as compared to the wild-type (Figure 5B, right panel). The dot plot showing the Pearson’s correlation coefficient for chromatin spread from the wild-type and the *zip1*∆ cells indicate most of the spreads in case of the mutant showed very less co-localization between Mam1 and Ndc10 (Figure S5). These results indicate that lack of *Zip1* at the centromere limits Mam1 localization at the centromere which in turn causes mono-orientation defect.

## Discussion

Mono-orientation of the sister kinetochores with respect to the meiotic spindle is of immense significance as this is one of the key events that lead to essential reductional division during meiosis I. The mechanism of this crucial event is poorly understood in higher systems. However, in budding yeast, a dedicated complex, called monopolin is directly involved in having the sister kinetochores co-oriented so that both can attach to the microtubules emanating from a single spindle pole (Rabitsch *et al.* 2003). The monopolin is believed to work as a clamp that puts the two sister kinetochores together constraining them to take side-by-side positions which facilitates their co-orientation (Corbett *et al.* 2010; Sarangapani *et al.* 2014). Therefore, any factor that can favor a side-by-side positioning would principally promote monopolin to achieve sister kinetochore mono-orientation. Such a factor might promote mono-orientation just by constraining the sister centromeres/kinetochores and favoring their side-by-side geometry congenial for unipolar spindle microtubule attachment. The function of condensin in sister mono-orientation is believed to follow this notion in a monopolin dependent way (Brito *et al.* 2010; Tada *et al.* 2011; Burrack *et al.* 2013). However, limiting rotation freedom could also impede proper orientation if the kinetochores are prevented from turning in the same direction. The ability of *Zip1* to couple the centromeres (Tsubouchi and Roeder 2005), its role in cross-linking proteinaceous structure during SC assembly (Sym *et al.* 1993) and its extended localization at the centromere during kinetochore-microtubule attachment beyond SC disassembly (Gladstone *et al.* 2009), have encouraged us to hypothesize that *Zip1* may be another factor that may constrain the sister centromeres to take side-by-side positions and thus *Zip1* might facilitate mono-orientation of the sister kinetochores. In this work, we tested this hypothesis and found that *Zip1* indeed has a moderate role in the process perhaps by stabilizing the monopolin at the centromere. 

A reduction in sporulation efficiency (Figure 1B) and spore viability (Figure 1A and E) in *zip1*Δ is expected as SC formation is disrupted and crossing over frequency is reduced (Sym *et al.* 1993). Interestingly, we noticed more than 40% dyads in *zip1*Δ cells compared to less than 5% in the wild-type (Figure 1D) which was encouraging as the appearance of dyads is suggestive of abrogation of one nuclear division which may occur if meiosis I cannot take place due to suppression of mono-orientation. As expected dyads were generated in *mam1*Δ cells as well that were compromised in monopolin function (Figure 2C). However, the percentage of dyads was less in *mam1*Δ than in *zip1*Δ cells (Figure 1D). This is probably because although meiosis I is abrogated in most of the *mam1*Δ cells, some cells go ahead till the end of meiosis II producing four spores where two spores hardly contain any DNA (Tóth *et al.* 2000). Nevertheless, the formation of dyads gave a hint that in *zip1*∆ mutant mono-orientation may be affected. 

It is important to address whether the observed role of *Zip1* in mono-orientation of the sisters (Figure 2D, 3C) is independent of its function in SC formation. To answer this, bi-orientation frequency was measured in the *zip1*∆ and the *mam1*∆ cells in the absence of Spo11 and hence SC. The ratio of this frequency between *zip1*∆ and *mam1*∆ cells (1:2, Figure 3C) remains almost similar to what has been observed between *spo11*∆ *zip1*∆ and *spo11*∆ *mam1*∆ cells (1:2.6, Figure 4B). Further, the frequency was found more than three times higher in *spo11*∆ *zip1*∆ compared to *spo11*∆ alone (5% *vs.* 19%, Figure 4B) indicating that the role of *Zip1* in mono-orientation is independent of SC formation. We believe that it is rather the localization and function of *Zip1* at the centromere (Tsubouchi and Roeder 2005; Gladstone *et al.* 2009) that play a role in constraining the sister kinetochores to be co-oriented. In support of a targeted localization of *Zip1* to the centromere, a physical interaction between *Zip1* and the kinetochore protein, Nuf2 has been documented (Newman *et al.* 2000) which is reminiscent to the physical interaction of Mam1 with another kinetochore protein, Dsn1 in order to bind to the centromere (Newman *et al.* 2000; Sarkar *et al.* 2013).

To achieve mono-orientation, it is imperative that the rotational freedom of the sister kinetochores should be attenuated. They should be constrained such that they can form a side-by-side orientation that favors them to capture microtubules coming from the same spindle pole. The organisms with regional centromeres appear to use meiotic cohesin at the sister centromeres to conjoin them (Goldstein 1981; Li and Dawe 2009; Sakuno *et al.* 2009) in addition to other meiosis specific proteins such as Moa1 in fission yeast, functionally analogous to Spo13 of budding yeast (Yokobayashi and Watanabe 2005; Sakuno *et al.* 2009). In these organisms, a large heterochromatic pericentromere along with cohesin is believed to provide a structural rigidity defining the kinetochore architecture which is required for mono-orientation. Conversely, in budding yeast perhaps due to a point centromere and lack of pericentric heterochromatin, centromeric cohesin does not have any role in mono-orientation (Tóth *et al.* 2000); therefore, the necessary structural rigidity, in this organism, is provided by condensin that facilitates mono-orientation by augmenting monopolin function (Brito *et al.* 2010). Later similar observation was also made in *S. pombe* and in *C. albicans*, (Tada *et al.* 2011; Burrack *et al.* 2013). From these results, it is conceivable that any other factors besides cohesin and condensin that may physically constrain sister kinetochores in principle can aid in mono-orientation. In fission yeast, the linkage between the homologs in a bivalent (chiasmata) can resist bi-orientation of the sister kinetochores (Sakuno *et al.* 2011). Similarly, lack of chiasmata can lead to bi-orientation of the univalents in mouse and human (Kouznetsova *et al.* 2007; Nagaoka *et al.* 2011) whereas such a scenario has not been observed in budding yeast, worms or Arabidopsis (Klein *et al.* 1999; Chelysheva *et al.* 2005; Severson *et al.* 2009). Therefore, we speculated that there could be an additional factor present in the budding yeast that may provide geometric rigidity to the sister kinetochores required for their mono-orientation. The role of *Zip1* in cross-linking axial elements on the homologs, its role in coupling centromeres and importantly its SC-independent presence at the centromere at the right time, *i.e.*, during the kinetochore-microtubule attachment, makes us hypothesize that *Zip1* could be another such factor helping in constraining sister kinetochores. Additionally, from its role in the pairing of the centromeres, it has been demonstrated that *Zip1* promotes bi-orientation of the homologs on meiosis I spindle (Gladstone *et al.* 2009; Newnham *et al.* 2010). This function could potentially be a legacy of this protein’s role in mono-orientation of the sister kinetochores that we found, which is then eventually perceived as a role in bi-orientation of the homologs. It was important to address if *Zip1* facilitates mono-orientation in addition to the monopolin mediated pathway. However, we found no enhancement of the mono-orientation defect in the *zip1*∆ *mam1*∆ double mutant cells over the *mam1*∆ single mutant (Figure 3C) indicating that in this aspect *Zip1* works through Mam1. In support of this, we observed Mam1 localization at the centromere is perturbed in the absence of *Zip1* (Figure 5A). Notably, if the mono-orientation defect in *zip1*∆ is due to mislocalization of Mam1 from the centromere, the erroneous bi-orientation frequency (two split GFP dots) is expected to be similar for *zip1*∆ and *mam1*∆ cells which we failed to observe (Figure 3C). This is perhaps due to a difference in the outcomes between *zip1*∆ and *mam1*∆ cells with respect to the levels of Mam1 activity available at the centromere. In *zip1*Δ cells, some Mam1 remains at the centromere which is possibly sufficient to rescue the mono-orientation defect to some level. During pachytene, *Zip1* helps in the generation of a 100 nm wide structure (SC) at the chromosome axes to link the homologs, and it is reasonable to believe that this structure is maintained at the centromeres during metaphase I or at least at the time of kinetochore-microtubule attachment (Gladstone *et al.* 2009; Newnham *et al.* 2010). Here, the fact that the sister chromatids are closely held by condensin (Brito *et al.* 2010) and *Zip1* is likely holding homologs apart enough, maybe a crucial requirement for cross-linking the sister kinetochores by monopolin complex. In this scenario, the absence of *Zip1* will allow the homologs to come close enough so that monopolin may not be able to cross-link the sister centromeres leading to reduced Mam1 occupancy at the centromere (Figure 5A). However, mono-orientation of sister kinetochores maintained to some level (Figure 3C) may be via some feedback mechanisms which can allow the stabilized monopolin interaction at the centromere to some extent.

In this study, we provide evidence that *Zip1* has a moderate but significant role in mono-orientation of the sister kinetochores, a crucial phenomenon to achieve faithful meiosis. It appears that *Zip1* does this function by facilitating retention of Mam1 at the centromere. In budding yeast, localization of Mam1 at the centromere is a key event for sister chromatid mono-orientation. Given the importance of this process for the reductional division, it is not surprising that cells will adopt multiple mechanisms to reinforce this and thus *Zip1* may serve as another receptor, besides Dsn1, for Mam1 at the centromere. Thus this work advances the knowledge by revealing another layer of regulation of sister mono-orientation in the budding yeast. Our findings have strong general implications as the SC proteins are also found in the higher eukaryotes and hence may have more roles to play particularly when these organisms lack monopolin like dedicated complex. 

## References

[bib1] Adler J. Parmryd I., 2010 Quantifying colocalization by correlation: the Pearson correlation coefficient is superior to the Mander’s overlap coefficient. Cytometry A 77: 733–742. 10.1002/cyto.a.20896 20653013

[bib2] Agarwal M. Mehta G. Ghosh S. K., 2015 Role of Ctf3 and COMA subcomplexes in meiosis: Implication in maintaining Cse4 at the centromere and numeric spindle poles. Biochim. Biophys. Acta 1853: 671–684. 10.1016/j.bbamcr.2014.12.032 25562757

[bib3] Bailis J. M. Smith A. V. Roeder G. S., 2000 Bypass of a Meiotic Checkpoint by Overproduction of Meiotic Chromosomal Proteins. Mol. Cell. Biol. 20: 4838–4848. 10.1128/MCB.20.13.4838-4848.2000 10848609 PMC85935

[bib4] Bhuiyan H. Schmekel K., 2004 Meiotic chromosome synapsis in yeast can occur without spo11-induced DNA double-strand breaks. Genetics 168: 775–783. 10.1534/genetics.104.029660 15514052 PMC1448848

[bib5] Brito I. L. Yu H. G. Amon A., 2010 Condensins promote coorientation of sister chromatids during meiosis I in budding yeast. Genetics 185: 55–64. 10.1534/genetics.110.115139 20194961 PMC2870976

[bib6] Burrack L. S. Applen Clancey S. E. Chacon J. M. Gardner M. K. Berman J., 2013 Monopolin recruits condensin to organize centromere DNA and repetitive DNA sequences. Mol. Biol. Cell 24: 2807–2819. 10.1091/mbc.e13-05-0229 23885115 PMC3771944

[bib7] Chelysheva L. Diallo S. Vezon D. Gendrot G. Vrielynck N., 2005 AtREC8 and AtSCC3 are essential to the monopolar orientation of the kinetochores during meiosis. J. Cell Sci. 118: 4621–4632. 10.1242/jcs.02583 16176934

[bib8] Chen X. Suhandynata R. T. Sandhu R. Rockmill B. Mohibullah N., 2015 Phosphorylation of the Synaptonemal Complex Protein Zip1 Regulates the Crossover/Noncrossover Decision during Yeast Meiosis. PLoS Biol. 13: e1002329 10.1371/journal.pbio.1002329 26682552 PMC4684282

[bib9] Corbett K. D. Harrison S. C., 2012 Molecular architecture of the yeast monopolin complex. Cell Reports 1: 583–589. 10.1016/j.celrep.2012.05.012 22813733 PMC3494995

[bib10] Corbett K. D. Yip C. K. Ee L. S. Walz T. Amon A., 2010 The monopolin complex crosslinks kinetochore components to regulate chromosome-microtubule attachments. Cell 142: 556–567. 10.1016/j.cell.2010.07.017 20723757 PMC2955198

[bib11] Dong H. Roeder G. S., 2000 Organization of the yeast Zip1 protein within the central region of the synaptonemal complex. J. Cell Biol. 148: 417–426. 10.1083/jcb.148.3.417 10662769 PMC2174805

[bib12] Falk J. E. Chan A. C. Hoffmann E. Hochwagen A., 2010 A Mec1- and PP4-dependent checkpoint couples centromere pairing to meiotic recombination. Dev. Cell 19: 599–611. 10.1016/j.devcel.2010.09.006 20951350

[bib13] Gillett E. S. Espelin C. W. Sorger P. K., 2004 Spindle checkpoint proteins and chromosome-microtubule attachment in budding yeast. J. Cell Biol. 164: 535–546. 10.1083/jcb.200308100 14769859 PMC2171994

[bib14] Gladstone M. N. Obeso D. Chuong H. Dawson D. S., 2009 The synaptonemal complex protein Zip1 promotes bi-orientation of centromeres at meiosis I. PLoS Genet. 5: e1000771 10.1371/journal.pgen.1000771 20011112 PMC2781170

[bib15] Goldstein L. S., 1981 Kinetochore structure and its role in chromosome orientation during the first meiotic division in male D. melanogaster. Cell 25: 591–602. 10.1016/0092-8674(81)90167-7 6793236

[bib16] Henderson K. A. Keeney S., 2004 Tying synaptonemal complex initiation to the formation and programmed repair of DNA double-strand breaks. Proc. Natl. Acad. Sci. USA 101: 4519–4524. 10.1073/pnas.0400843101 15070750 PMC384779

[bib17] Hochwagen A. Wrobel G. Cartron M. Demougin P. Niederhauser-Wiederkehr C., 2005 Novel response to microtubule perturbation in meiosis. Mol. Cell. Biol. 25: 4767–4781. 10.1128/MCB.25.11.4767-4781.2005 15899877 PMC1140642

[bib18] Janke C. Magiera M. M. Rathfelder N. Taxis C. Reber S., 2004 A versatile toolbox for PCR-based tagging of yeast genes: new fluorescent proteins, more markers and promoter substitution cassettes. Yeast 21: 947–962. 10.1002/yea.1142 15334558

[bib19] Kiburz B. M. Amon A. Marston A. L., 2008 Shugoshin promotes sister kinetochore biorientation in Saccharomyces cerevisiae. Mol. Biol. Cell 19: 1199–1209. 10.1091/mbc.e07-06-0584 18094053 PMC2262988

[bib20] Klein F. Mahr P. Galova M. Buonomo S. B. Michaelis C., 1999 A central role for cohesins in sister chromatid cohesion, formation of axial elements, and recombination during yeast meiosis. Cell 98: 91–103. 10.1016/S0092-8674(00)80609-1 10412984

[bib21] Kouznetsova A. Lister L. Nordenskjold M. Herbert M. Hoog C., 2007 Bi-orientation of achiasmatic chromosomes in meiosis I oocytes contributes to aneuploidy in mice. Nat. Genet. 39: 966–968. 10.1038/ng2065 17618286

[bib22] Li X. Dawe R. K., 2009 Fused sister kinetochores initiate the reductional division in meiosis I. Nat. Cell Biol. 11: 1103–1108. 10.1038/ncb1923 19684578

[bib23] Longtine M. S. McKenzie A. 3rd Demarini D. J. Shah N. G. Wach A., 1998 Additional modules for versatile and economical PCR-based gene deletion and modification in Saccharomyces cerevisiae. Yeast 14: 953–961. 10.1002/(SICI)1097-0061(199807)14:10<953::AID-YEA293>3.0.CO;2-U 9717241

[bib24] Marston A. L. Amon A., 2004 Meiosis: cell-cycle controls shuffle and deal. Nat. Rev. Mol. Cell Biol. 5: 983–997. 10.1038/nrm1526 15573136

[bib25] Mehta G. D. Agarwal M. Ghosh S. K., 2014 Functional characterization of kinetochore protein, Ctf19 in meiosis I: an implication of differential impact of Ctf19 on the assembly of mitotic and meiotic kinetochores in Saccharomyces cerevisiae. Mol. Microbiol. 91: 1179–1199. 10.1111/mmi.12527 24446862

[bib26] Michaelis C. Ciosk R. Nasmyth K., 1997 Cohesins: chromosomal proteins that prevent premature separation of sister chromatids. Cell 91: 35–45. 10.1016/S0092-8674(01)80007-6 9335333

[bib27] Miller M. P. Unal E. Brar G. A. Amon A., 2012 Meiosis I chromosome segregation is established through regulation of microtubule-kinetochore interactions. eLife 1: e00117 10.7554/eLife.00117 23275833 PMC3525924

[bib28] Monje-Casas F. Prabhu V. R. Lee B. H. Boselli M. Amon A., 2007 Kinetochore orientation during meiosis is controlled by Aurora B and the monopolin complex. Cell 128: 477–490. 10.1016/j.cell.2006.12.040 17289568 PMC1808280

[bib29] Nagaoka S. I. Hodges C. A. Albertini D. F. Hunt P. A., 2011 Oocyte-specific differences in cell-cycle control create an innate susceptibility to meiotic errors. Curr. Biol. 21: 651–657. 10.1016/j.cub.2011.03.003 21497085 PMC3225230

[bib30] Neiman A. M., 2005 Ascospore Formation in the Yeast Saccharomyces cerevisiae. Microbiol. Mol. Biol. Rev. 69: 565–584. 10.1128/MMBR.69.4.565-584.2005 16339736 PMC1306807

[bib31] Newman J. R. Wolf E. Kim P. S., 2000 A computationally directed screen identifying interacting coiled coils from Saccharomyces cerevisiae. Proc. Natl. Acad. Sci. USA 97: 13203–13208. 10.1073/pnas.97.24.13203 11087867 PMC27203

[bib32] Newnham L. Jordan P. Rockmill B. Roeder G. S. Hoffmann E., 2010 The synaptonemal complex protein, Zip1, promotes the segregation of nonexchange chromosomes at meiosis I. Proc. Natl. Acad. Sci. USA 107: 781–785. 10.1073/pnas.0913435107 20080752 PMC2818913

[bib33] Obeso D. Dawson D. S., 2010 Temporal characterization of homology-independent centromere coupling in meiotic prophase. PLoS One 5: e10336 10.1371/journal.pone.0010336 20428251 PMC2859069

[bib34] Petronczki M. Matos J. Mori S. Gregan J. Bogdanova A., 2006 Monopolar attachment of sister kinetochores at meiosis I requires casein kinase 1. Cell 126: 1049–1064. 10.1016/j.cell.2006.07.029 16990132

[bib35] Prajapati H. K. Rizvi S. M. Rathore I. Ghosh S. K., 2017 Microtubule-associated proteins, Bik1 and Bim1, are required for faithful partitioning of the endogenous 2 micron plasmids in budding yeast. Mol. Microbiol. 103: 1046–1064. 10.1111/mmi.13608 28004422

[bib36] Rabitsch K. P. Petronczki M. Javerzat J. P. Genier S. Chwalla B., 2003 Kinetochore recruitment of two nucleolar proteins is required for homolog segregation in meiosis I. Dev. Cell 4: 535–548. 10.1016/S1534-5807(03)00086-8 12689592

[bib37] Rutkowski L. H. Esposito R. E., 2000 Recombination can partially substitute for SPO13 in regulating meiosis I in budding yeast. Genetics 155: 1607–1621. 10924460 10.1093/genetics/155.4.1607 PMC1461194

[bib38] Sakuno T. Tada K. Watanabe Y., 2009 Kinetochore geometry defined by cohesion within the centromere. Nature 458: 852–858. 10.1038/nature07876 19370027

[bib39] Sakuno T. Tanaka K. Hauf S. Watanabe Y., 2011 Repositioning of Aurora B Promoted by Chiasmata Ensures Sister Chromatid Mono-Orientation in Meiosis I. Dev. Cell 21: 534–545. 10.1016/j.devcel.2011.08.012 21920317

[bib40] Sarangapani K. K. Duro E. Deng Y. Alves F. L. Ye Q., 2014 Sister kinetochores are mechanically fused during meiosis I in yeast. Science 346: 248–251. 10.1126/science.1256729 25213378 PMC4226495

[bib41] Sarkar S. Shenoy R. T. Dalgaard J. Z. Newnham L. Hoffmann E., 2013 Monopolin subunit Csm1 associates with MIND complex to establish monopolar attachment of sister kinetochores at meiosis I. PLoS Genet. 9: e1003610 10.1371/journal.pgen.1003610 23861669 PMC3701701

[bib42] Severson A. F. Ling L. van Zuylen V. Meyer B. J., 2009 The axial element protein HTP-3 promotes cohesin loading and meiotic axis assembly in C. elegans to implement the meiotic program of chromosome segregation. Genes Dev. 23: 1763–1778. 10.1101/gad.1808809 19574299 PMC2720254

[bib43] Sym M. Engebrecht J. A. Roeder G. S., 1993 ZIP1 is a synaptonemal complex protein required for meiotic chromosome synapsis. Cell 72: 365–378. 10.1016/0092-8674(93)90114-6 7916652

[bib44] Sym M. Roeder G. S., 1994 Crossover interference is abolished in the absence of a synaptonemal complex protein. Cell 79: 283–292. 10.1016/0092-8674(94)90197-X 7954796

[bib45] Sym M. Roeder G. S., 1995 Zip1-induced changes in synaptonemal complex structure and polycomplex assembly. J. Cell Biol. 128: 455–466. 10.1083/jcb.128.4.455 7860625 PMC2199901

[bib46] Tada K. Susumu H. Sakuno T. Watanabe Y., 2011 Condensin association with histone H2A shapes mitotic chromosomes. Nature 474: 477–483. 10.1038/nature10179 21633354

[bib47] Thacker D. Mohibullah N. Zhu X. Keeney S., 2014 Homologue engagement controls meiotic DNA break number and distribution. Nature 510: 241–246. 10.1038/nature13120 24717437 PMC4057310

[bib48] Tóth A. Rabitsch K. P. Galova M. Schleiffer A. Buonomo S. B., 2000 Functional genomics identifies monopolin: a kinetochore protein required for segregation of homologs during meiosis i. Cell 103: 1155–1168. 10.1016/S0092-8674(00)00217-8 11163190

[bib49] Tsubouchi T. Roeder G. S., 2005 A synaptonemal complex protein promotes homology-independent centromere coupling. Science 308: 870–873. 10.1126/science.1108283 15879219

[bib50] Verzijlbergen K. F. Nerusheva O. O. Kelly D. Kerr A. Clift D., 2014 Shugoshin biases chromosomes for biorientation through condensin recruitment to the pericentromere. eLife 3: e01374 10.7554/eLife.01374 24497542 PMC3910079

[bib51] Yokobayashi S. Watanabe Y., 2005 The kinetochore protein Moa1 enables cohesion-mediated monopolar attachment at meiosis I. Cell 123: 803–817. 10.1016/j.cell.2005.09.013 16325576

[bib52] Zinchuk V. Zinchuk O., 2008 Quantitative colocalization analysis of confocal fluorescence microscopy images. Curr. Protoc. Cell Biol. Chapter 4: Unit 4.19 10.1002/0471143030.cb0419s39 18551422

